# Hypertension and Polycystic Ovary Syndrome Among Women in a Nationwide Electronic Health Records Dataset in the United States

**DOI:** 10.1007/s10995-025-04155-x

**Published:** 2025-09-01

**Authors:** Siran He, Omoye Imoisili, Lyudmyla Kompaniyets, Elizabeth A. Lundeen, Elena V. Kuklina, Sandra L. Jackson

**Affiliations:** 1 Division for Heart Disease and Stroke Prevention, National Center for Chronic Disease Prevention and Health Promotion, Centers for Disease Control and Prevention, Atlanta, Georgia; 2 Division of Nutrition, Physical Activity, and Obesity, National Center for Chronic Disease Prevention and Health Promotion, Centers for Disease Control and Prevention, Atlanta, Georgia

**Keywords:** Polycystic ovary syndrome, Hypertension, Cardiovascular disease, Women’s health, Electronic health records

## Abstract

**Introduction:**

Both hypertension and polycystic ovary syndrome (PCOS) are risk factors for future cardiovascular diseases among women of reproductive age (18–44 years). We constructed an electronic health record (EHR)-based PCOS phenotype, reported PCOS prevalence, and investigated the association of PCOS and hypertension in the United States (US).

**Methods:**

This cross-sectional study used 2022 IQVIA’s Ambulatory Electronic Medical Record (AEMR)-US data (May 2023 release). We constructed a phenotype for PCOS and reported PCOS prevalence for eligible women. We then described hypertension prevalence and hypertension control estimates stratified by PCOS status. Lastly, we calculated adjusted prevalence ratios (aPR) for hypertension and hypertension control by PCOS status, adjusting for age, race, and body mass index (BMI).

**Results:**

We analyzed records for 1,301,425 eligible women, with mean (standard deviation) age of 31.5 (7.9) years. The prevalence of PCOS was 2.1%, but increased with weight category, reaching 6.7% among those with class 3 obesity (BMI ≥ 40 kg/m^2^). Women with PCOS had 50% higher prevalence of hypertension than those without PCOS (aPR 1.50; 95% confidence interval [CI]: 1.48–1.52; *p* < 0.001), and slightly higher hypertension control prevalence (aPR 1.14; 95% CI: 1.12–1.17; *p* < 0.001).

**Discussion:**

Using a nationwide EHR dataset, we observed that women with PCOS had substantially higher hypertension prevalence than those without PCOS. PCOS prevalence was lower than previous estimates from global surveys. Following guideline-recommended blood pressure screening for women with PCOS could reduce the risk of long-term cardiovascular disease.

**Supplementary Information:**

The online version contains supplementary material available at 10.1007/s10995-025-04155-x.

## Significance

What is Already Known on this Subject??

What this Study Adds??

Polycystic ovary syndrome (PCOS) and hypertension both contribute to future cardiovascular diseases among women of reproductive age. PCOS is also a known risk factor for hypertension. Information is lacking on PCOS prevalence and its association with hypertension in the United States (US).

We developed an electronic health record (EHR)-based phenotype for PCOS, and estimated PCOS prevalence in the US. We also observed that women with PCOS had 50% higher hypertension than those without. Women of reproductive age with PCOS should be screened for high blood pressure to promote long-term cardiovascular health.

**Supplementary Information:**

The online version contains supplementary material available at 10.1007/s10995-025-04155-x.

## Introduction

Polycystic ovary syndrome (PCOS) is an endocrine disorder with complex contribution factors (e.g., genetic, environmental, metabolic) and varying clinical and subclinical manifestations (Actkins et al., 2021). Globally, PCOS prevalence is 10–13%, but 75% of cases may go undiagnosed in the United States (US) (Christ & Cedars, 2023; Teede et al., 2023). Prior research has largely focused on the negative impact of PCOS on reproductive health, including infertility, gestational diabetes, miscarriage, and even perinatal death. (American College of Obstetricians and Gynecologists’ Committee on Practice Bulletins—Gynecology, 2018; Mills et al., 2020; National Institutes of Health, 2012; Yu et al., 2016). In contrast, less attention has been paid to the risk PCOS poses beyond reproductive health: PCOS is associated with cardiometabolic disturbances including insulin resistance, hypertension, and cardiovascular disease (CVD) (Osibogun et al., 2020).

Recent research indicates that women of reproductive age with PCOS have a 1.7-fold higher risk for hypertension than those without PCOS (Amiri et al., 2020), therefore PCOS and hypertension can have compounded negative impact on women’s long-term CVD health. However, there is a lack of US public health surveillance data on PCOS prevalence and its association with hypertension. In this study, we aimed to develop an electronic health record (EHR)-based phenotype for PCOS to estimate PCOS prevalence, and to analyze the prevalence of hypertension and hypertension control by PCOS status among women of reproductive age.

## Methods

### Population

For this cross-sectional study we used the IQVIA’s Ambulatory Electronic Medical Records-US (AEMR-US) data, May 2023 release. The AEMR-US data used in this study was in the Observational Medical Outcomes Partnership (OMOP v5.3) format, which is a common data model with standardized domains and data elements. This dataset covered over 100,000 outpatient care providers, with data from patients in 50 states and the District of Columbia.

**Fig. 1:**
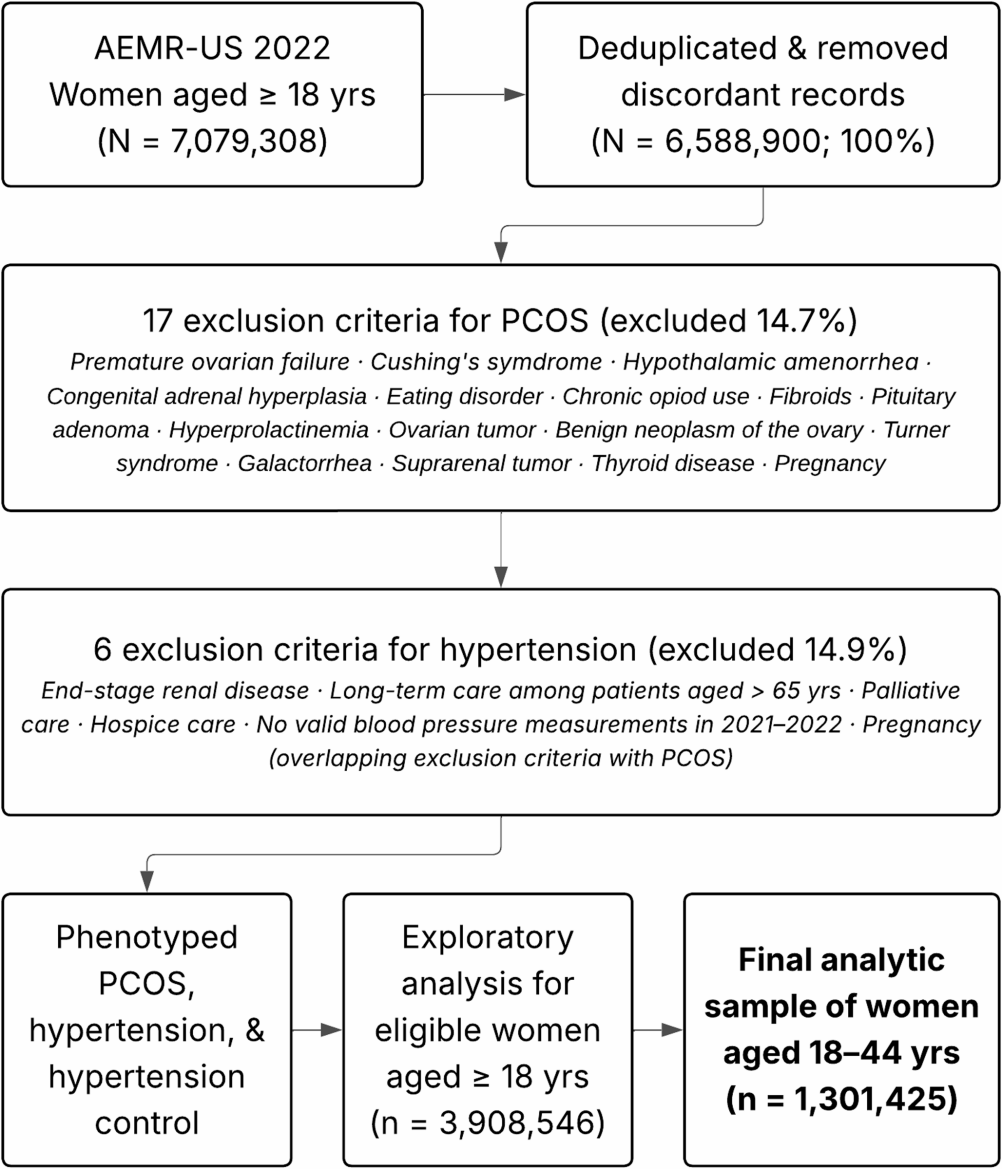
Flow chart of the study population. AEMR, IQVIA ambulatory electronic medical record-US; PCOS, polycystic ovary syndrome

We included adult women with at least one outpatient encounter in 2022. After deduplication and removing discordant records (i.e., records with multiple sexes, years of births, and race or ethnicity), there were 6,588,900 women. We applied two sets of exclusion criteria (Fig. 1). PCOS-related exclusions (14.7%), which could mimic or mask symptoms of PCOS, were adapted from a previous phenotype (Actkins et al., 2021). We refined this phenotype through a clinician-guided process, and excluded women who had any of the following: premature ovarian failure, pregnancy, Cushing’s syndrome, hypothalamic amenorrhea, congenital adrenal hyperplasia, eating disorder, chronic opioid use, fibroids, pituitary adenoma, hyperprolactinemia, ovarian tumor, benign neoplasm of the ovary, Turner syndrome, galactorrhea, suprarenal tumor, and thyroid disease. Subsequently, hypertension-related exclusions followed an electronic clinical quality measure, CMS165 “Controlling High Blood Pressure” (https://ecqi.healthit.gov/ecqm/ec/2023/cms0165v11?qt-tabs_measure=measure-information). Patients were excluded if they had end-stage renal disease, were in palliative care or hospice care, or were in long-term care (among patients aged > 65 years). We also excluded patients who did not have at least one valid blood pressure (BP) measurement in the past 24 months. We restricted the final analysis to 1,301,425 women aged 18–44 years.

## Measures

The three key measures in this study were PCOS, hypertension, and hypertension control status. We developed a PCOS phenotype using the Rotterdam Criteria (National Institutes of Health, 2012). We defined PCOS as having a PCOS diagnosis, which referred to International Classification of Diseases 10th Revision Clinical Modification (ICD-10-CM) code E28.2; or having at least two out of the following three criteria during 2022: (i) polycystic ovary, ICD-9-CM code 256.4, and ICD-10-CM codes N83.2* (the asterisk denotes that all ICD codes with this prefix was included); (ii) oligo- or anovulation, ICD-9-CM codes 626.* (excluding 626.2, 626.6, and 626.7), and ICD-10-CM codes N91.*, N92.5 and N92.6; (iii) hyperandrogenism, ICD-9-CM code 704.1, and ICD-10-CM codes L68.0 and E28.1. In addition, we defined hypertension as having any of the following during 2022: at least one diagnosis code, at least two BP measurements ≥ 130/80 mm Hg, or at least one antihypertensive medication, as described elsewhere (He et al., 2023, 2024). Among those with hypertension, we defined hypertension control as having the most recent BP measurement in 2022 lower than 130/80 mm Hg (He et al., 2023).

### Statistical Analysis

Among all eligible women, we described the distribution of age, race (Asian, Black, White, other), body mass index (BMI in kg/m^2^), weight categories (underweight, BMI < 18.5; healthy weight, BMI ≥ 18.5 to < 25.0; overweight, BMI ≥ 25.0 to < 30.0; obesity, BMI ≥ 30, with three BMI-based sub-classes: https://www.cdc.gov/bmi/adult-calculator/bmi-categories.html), type of health care provider, and relevant biomarkers. We used Student’s *t*-test, Mann-Whitney *U* test, or χ^2^ test for comparisons by PCOS status. We reported crude PCOS prevalence—total, and stratified by age, race, and weight category. Furthermore, we reported crude hypertension and hypertension control prevalence by PCOS status, and χ^2^ test was used for comparison. We analyzed associations between PCOS and hypertension prevalence and control, adjusting for covariates, from logistic regression models and calculating adjusted prevalence ratios (aPR) through marginal standardization (delta method for standard errors). We determined statistical significance a priori at *p* < 0.05, and used R (version 4.2.3; R Core Group, Vienna, Austria) for all analyses, conducted during October 2023–January 2024. The Centers for Disease Control and Prevention (CDC) reviewed this activity, conducted consistent with applicable federal law and CDC policy.^1^

## Results

**Fig. 2:**
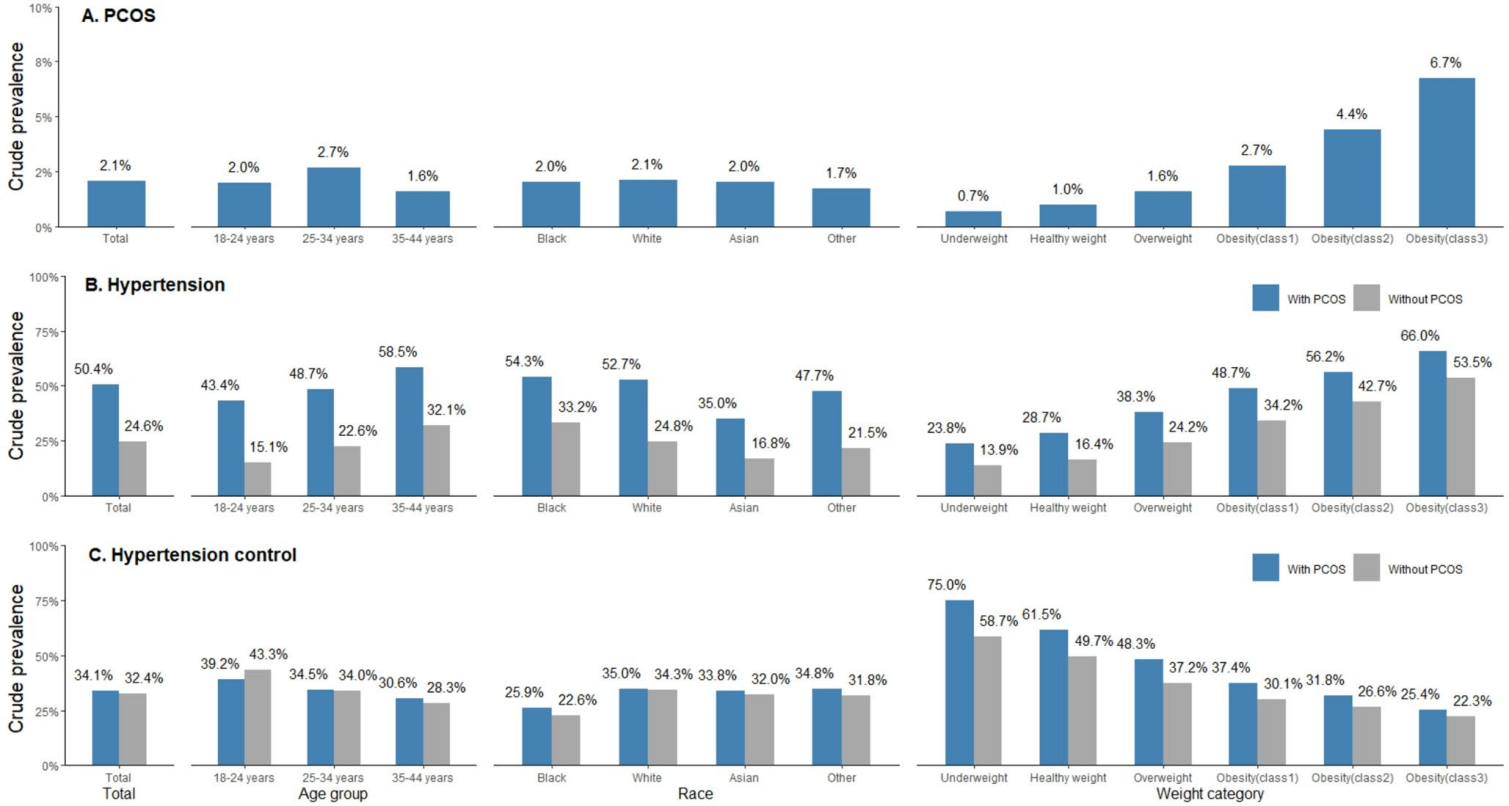
Prevalence of polycystic ovary syndrome, hypertension, and hypertension control in AEMR-US 2022. IQVIA combines race and ethnicity in the same field. Data shown may not reflect the true racial and ethnic information. “Other” category refers to Hispanic ethnicity, multiracial, and other racial and ethnic individuals. Weight categories: underweight, BMI < 18.5 kg/m^2^; healthy weight, BMI ≥ 18.5 to < 25.0 kg/m^2^; overweight, BMI ≥ 25.0 to < 30.0 kg/m^2^; obesity class 1, BMI ≥ 30.0 to < 35.0 kg/m^2^; obesity class 2, BMI ≥ 35.0 to < 40.0 kg/m^2^; obesity class 3, BMI ≥ 40.0 kg/m^2^. AEMR-US, IQVIA Ambulatory Electronic Medical Record-US; PCOS, polycystic ovary syndrome

Among 1.3 million women aged 18–44 years in the analysis, crude PCOS prevalence was 2.1%, ranging from 0.7% among women classified as underweight to 6.7% among those who had class 3 obesity (Fig. 2, Panel A). More women with PCOS had obesity than those without PCOS (65.9% vs. 30.5%; *p* < 0.001), particularly class 3 obesity (29.7% vs. 8.6%) (Table 1). Women with PCOS also had higher measurements of systolic and diastolic BP (SBP, DBP), total and free testosterone, fasting insulin, triglycerides, and low-density lipoprotein cholesterol, but lower high-density lipoprotein cholesterol compared to women without PCOS (all *p* < 0.01).

**Table 1:** Characteristics of the study population in AEMR-US, 2022

Characteristics	All eligible women (*N* = 1,301,425)		Without PCOS (*n* = 1,274,574)		With PCOS (*n* = 26,851)		*p*-value^a^
	*n*	% or Mean (SD)	*n*	% or Mean (SD)	*n*	% or Mean (SD)	
**Hypertension**	327,136	25.1%	313,591	24.6%	13,545	50.4%	< 0.001
**Hypertension control**	106,170	32.5%	101,555	32.4%	4,615	34.1%	< 0.001
**Age**,** year**,** mean (SD)**	1,301,425	31.5 (7.9)	1,274,574	31.6 (7.9)	26,851	30.5 (7.0)	< 0.001
**Age groups**,** %**,							< 0.001
18–24 years	320,984	24.7%	314,605	24.7%	6,379	23.8%	
25–34 years	455,383	35.0%	443,192	34.8%	12,191	45.4%	
35–44 years	525,058	40.3%	516,777	40.5%	8,281	30.8%	
Total	1,301,425	100.0%	1,274,574	100.0%	26,851	100.0%	
**Race**,^**b**^ **%**							< 0.001
White	808,726	62.1%	791,710	62.1%	17,016	63.4%	
Black	120,254	9.2%	117,852	9.2%	2,402	8.9%	
Asian	32,681	2.5%	32,022	2.5%	659	2.5%	
Other	37,928	2.9%	37,278	2.9%	650	2.4%	
Unknown	301,836	23.2%	295,712	23.2%	6,124	22.8%	
Total	1,301,425	100.0%	1,274,574	100.0%	26,851	100.0%	
**Health care provider type**, **%**							< 0.001
Primary care	396,053	30.4%	387,663	30.4%	8,390	31.2%	
Reproductive health	56,130	4.3%	53,671	4.2%	2,459	9.2%	
Endocrinology	2,672	0.2%	2,410	0.2%	262	1.0%	
Cardiovascular disease	11,472	0.9%	11,321	0.9%	151	0.6%	
Other	653,880	50.2%	641,861	50.4%	12,019	44.8%	
Unknown	181,218	13.9%	177,648	13.9%	3,570	13.3%	
Total	1,301,425	100.0%	1,274,574	100.0%	26,851	100.0%	
**Anthropometric measurements**							
BMI, kg/m^2^, mean (SD)	1,036,863	29.3 (7.9)	1,011,587	29.2 (7.9)	25,276	35.4 (8.8)	< 0.001
Weight category, %							< 0.001
Underweight	24,396	1.9%	24,228	1.9%	168	0.6%	
Healthy weight	348,575	26.8%	345,230	27.1%	3,345	12.5%	
Overweight	257,744	19.8%	253,675	19.9%	4,069	15.2%	
Obesity–combined	406,148	31.2%	388,454	30.5%	17,694	65.9%	
Obesity class 1	175,972	13.5%	171,157	13.4%	4,815	17.9%	
Obesity class 2	111,976	8.6%	107,064	8.4%	4,912	18.3%	
Obesity class 3	118,200	9.1%	110,233	8.6%	7,967	29.7%	
Unknown	264,562	20.3%	262,987	20.6%	1,575	5.9%	
Total	1,301,425	100.0%	1,274,574	100.0%	26,851	100.0%	
**Biomarkers**,** mean (SD)**							
Systolic BP, mm Hg	1,106,583	118.9 (12.3)	1,080,355	118.8 (12.3)	26,228	121.6 (11.4)	< 0.001
Diastolic BP, mm Hg	1,106,583	75.4 (9.0)	1,080,355	75.4 (9.0)	26,228	77.4 (8.1)	< 0.001
Total testosterone, ng/dL	8,570	36.2 (32.1)	6,462	33.3 (34.3)	2,108	42.6 (25.6)	< 0.001
Free testosterone, ng/dL	3,037	0.4 (0.5)	2,210	0.3 (0.5)	827	0.6 (0.4)	< 0.001
Free androgen index	1,432	3.8 (4.4)	997	3.0 (3.7)	435	5.7 (5.2)	< 0.001
Fasting insulin, IU/mL	517	25.3 (30.7)	374	22.2 (25.7)	134	33.4 (39.8)	0.002
Fasting glucose, mg/dL	3,385	99.1 (43.9)	3,232	99.1 (44.5)	153	99.3 (28.8)	0.954
Total cholesterol, mg/dL	222,631	183.2 (35.4)	213,485	183.1 (35.4)	9,146	185.7 (35.9)	< 0.001
Triglycerides, mg/dL	242,606	109.4 (73.2)	232,863	108.4 (72.8)	9,743	133.9 (79.1)	< 0.001
HDL cholesterol, mg/dL	174,993	56.9 (15.3)	168,183	57.2 (15.3)	6,810	51.1 (13.7)	< 0.001
LDL cholesterol, mg/dL	190,222	106.0 (31.0)	182,601	105.8 (30.9)	7,621	110.2 (32.0)	< 0.001

^a^ Comparisons between women with and without PCOS were done using Student’s *t*-test, Mann–Whitney *U* Test, or χ2 test depending on the type and distribution of the variable.

^b^ IQVIA combines race and ethnicity in the same field. Data shown may not reflect the true racial and ethnic information. “Other” category refers to Hispanic ethnicity, multiracial, and other racial and ethnic individuals.

AEMR-US, IQVIA Ambulatory Electronic Medical Record-US; BMI, body mass index; BP, blood pressure; HDL, high-density lipoprotein; LDL, low-density lipoprotein; PCOS, polycystic ovary syndrome; SD, standard deviation.

Overall, women of reproductive age with PCOS had higher hypertension prevalence than those without PCOS (50.4% vs. 24.6%; *p* < 0.001), and this difference held true across age, race, and weight strata (Fig. 2, Panel B). After adjusting for age, BMI, and race, women with PCOS had 50% higher prevalence of hypertension than those without PCOS (aPR 1.50; 95% confidence interval [CI]: 1.48–1.52; *p* < 0.001) (Table 2). Among women identified as having hypertension, hypertension control estimates were similar between women with and without PCOS (34.1% and 32.4%, respectively), and decreased as weight category went up (Fig. 2, Panel C). After holding other covariates constant, hypertension control estimates were higher among women with PCOS than without (aPR 1.14; 95% CI: 1.12–1.17; *p* < 0.001) (Table 2).

**Table 2:** Hypertension and hypertension control prevalence ratio by polycystic ovary syndrome status in AEMR-US, 2022

Model parameters	Hypertension ^a^	Hypertension control ^b^
	aPR (95% CI) ^c^	aPR (95% CI) ^c^
**Exposure variable**		
Without PCOS	*Referent*	*Referent*
With PCOS	1.50 (1.48, 1.52)	1.14 (1.12, 1.17)
**Covariates**:		
*Age*,* year*	1.04 (1.04, 1.04)	0.99 (0.99, 0.99)
*BMI*,* kg/m*^*2*^	1.07 (1.07, 1.07)	0.99 (0.99, 0.99)
*Race*		
Asian	*Referent*	*Referent*
Black	1.37 (1.34, 1.40)	0.91 (0.88, 0.95)
White	1.25 (1.22, 1.28)	1.17 (1.12, 1.21)
Other	1.07 (1.04, 1.09)	1.12 (1.07, 1.17)

^a^Sample size for hypertension-related regression model: Complete case analysis *N* = 1,036,863; hypertension = 297,660; PCOS = 25,276

^b^Sample size for hypertension control-related regression model: Complete case analysis = 297,660; hypertension controlled = 100,387; PCOS = 12,917

^c^All *P* values were < 0.001

AEMR-US, IQVIA Ambulatory Electronic Medical Record-US; aPR, adjusted prevalence ratio; BMI, body mass index; CI, confidence interval; PCOS, polycystic ovary syndrome

## Discussion

To our knowledge, this is the first study to investigate the association between PCOS and hypertension among women in a nationwide EHR dataset in the US. Women of reproductive age with PCOS had 50% higher hypertension prevalence than those without PCOS, but comparable, suboptimal hypertension control rates. We observed a PCOS prevalence of 2% in the US, which was lower than estimated global prevalence of 10–13% (Teede et al., 2023).

Our findings underscore the importance of hypertension screening and management for women with PCOS, among whom CVD may manifest during reproductive years and can persist through the peri- and post-menopausal years (National Institutes of Health, 2012). Women with PCOS had 2.8 mmHg higher SBP than women without PCOS, and even modest differences in BP can have clinically meaningful cardiovascular effects. For example, a 5 mmHg decline in SBP has been associated with 10% reduction in cardiovascular outcomes (Canoy et al., 2022). Early prevention, screening, and management of hypertension among women with PCOS may therefore have long-lasting protective effects (Teede et al., 2023). The 2023 International Evidence-Based Guideline for PCOS (hereafter referred to as the 2023 PCOS Guideline) recommends assessing CVD risk factors in all women with PCOS, including measuring BP annually and during pregnancy or infertility treatment (Teede et al., 2023). This guideline further proposed the inclusion of PCOS as a risk factor in general population guidelines for CVD (Teede et al., 2023).

The underestimation of PCOS prevalence is not surprising, given the diverse symptoms (Christ & Cedars, 2023). The Rotterdam Criteria include polycystic ovarian morphology as a possible criterion, which requires transvaginal ultrasound that may be a barrier for some women (although the 2023 PCOS Guideline included serum anti-Müllerian hormone as an alternative to ultrasound in adults) (Legro et al., 2013; Teede et al., 2023). A 2015–2016 survey also revealed knowledge gaps among gynecologists and reproductive endocrinologists: one-third of respondents did not know which PCOS criteria to use (Dokras et al., 2017). Lack of standardization in diagnostic criteria may have contributed to this issue (Skiba et al., 2018). In addition, providers might not be reimbursed for their services related to PCOS if interpreted as infertility-related by payers (“Ob-Gyn Coding Alert 2003 Newsletter: Take Steps to Get PCOS Diagnosis Paid,” 2003). Removing reimbursement barriers may improve PCOS coding and diagnosis rates.

This study has limitations. First, the nature of EHR data can introduce selection bias, as these data reflect a care-seeking population, which may have intrinsic differences from the general population. Second, we excluded pregnant women from the study population. Although PCOS is related to difficulty becoming pregnant, as well as adverse pregnancy outcomes after conception, pregnancy itself had to be ruled out for proper PCOS diagnosis. (Williams et al., 2016) Third, partly due to the high degree of missingness of key biomarkers related to PCOS, we relied on diagnosis codes to identify PCOS, potentially leading to underestimation of PCOS prevalence. Fourth, one component of our hypertension definition was antihypertensive medication, including spironolactone, which can also be used “off-label” for PCOS symptom management. If some women with PCOS were prescribed spironolactone without having hypertension, hypertension prevalence could have been overestimated. A more conservative estimate of hypertension prevalence among women with PCOS was 43.0% (Online Resource 1), after excluding spironolactone from the analysis. Lastly, we were unable to investigate the role of key biomarkers, such as testosterone, in the association between PCOS and hypertension. Future research is needed to further investigate relevant biochemical pathways within the context of population science.

Through this EHR-based study, we filled important gaps in chronic disease surveillance. We observed a strong association between PCOS and hypertension among women aged 18–44 years in the US. We also observed lower-than-expected PCOS prevalence, which could reflect under-diagnosis of PCOS in US healthcare systems, and potentially suggests the need for alternative data sources (such as nationally representative surveys) to more sensitively capture PCOS..

Our findings align with the 2023 PCOS Guideline, which recommended annual BP screening for women with PCOS (Teede et al., 2023). Improving BP screening and hypertension treatment among women of reproductive age who have PCOS can help protect their long-term CVD health.

## Supplementary Information

Below is the link to the electronic supplementary material.

Supplementary material 1 (PDF 85.9 kb)

## Data Availability

The data underlying this article are proprietary and cannot be shared publicly.

## Footnotes

1 45 C.F.R. part 46, 21 C.F.R. part 56; 42 U.S.C. Section 241(d); 5 U.S.C. Sect. 552a; 44 U.S.C. Sect. Section 3501 et seq.

## Code Availability

Standard codes for the e-phenotypes will be shared upon reasonable request to the corresponding author.
