# Supplementary material for: Hypertension and Polycystic Ovary Syndrome Among Women in a Nationwide Electronic Health Records Dataset in the United States
Source: Matern Child Health J. 2025 Sep 1;29(10):1352–9. doi: 10.1007/s10995-025-04155-x (PMC12484343; doi:10.1007/s10995-025-04155-x)
Supplement: Supplementary file 1 — Supplementary material 1 (PDF 85.9 kb) [file 10995_2025_4155_MOESM1_ESM.pdf]

Electronic Supplementary Material

Title: Hypertension and Polycystic Ovary Syndrome Among Women in a Nationwide Electronic Health Records Dataset in the United States

Journal name: Maternal and Child Health Journal

Authors: Siran He[1], PhD, MSPH, Omoye Imoisili[1], MD, MPH, Lyudmyla Kompaniyets[2], PhD, MS, MS, Elizabeth A. Lundeen[1], PhD, MPH, Elena V. Kuklina[1], MD, PhD, Sandra L. Jackson[1], PhD, MPH

Affiliations:

[1] Division for Heart Disease and Stroke Prevention, National Center for Chronic Disease Prevention and Health Promotion, Centers for Disease Control and Prevention, Atlanta, Georgia

[2] Division of Nutrition, Physical Activity, and Obesity, National Center for Chronic Disease Prevention and Health Promotion, Centers for Disease Control and Prevention, Atlanta, Georgia

Online Resource 1. Sensitivity analysis for hypertension case identification, with and without spironolactone, stratified by PCOS status in AEMR-US, 2022

Hypertension criteria [a]	All eligible women (N = 1,301,425)		Without PCOS (n = 1,274,574)		With PCOS (n = 26,851)	
	n	%	n	%	n	%
**With spironolactone [b]**						
Diagnosis code only	8,337	0.6%	8,090	0.6%	247	0.9%
High BP only	135,316	10.4%	130,174	10.2%	5,142	19.2%
Medication only	86,681	6.7%	83,497	6.6%	3,184	11.9%
Any criteria	327,136	25.1%	313,591	24.6%	13,545	50.4%
**Without spironolactone**						
Diagnosis code only	8,639	0.7%	8,339	0.7%	300	1.1%
High BP only	139,541	10.7%	133,517	10.5%	6,024	22.4%
Medication only	63,853	4.9%	62,673	4.9%	1,180	4.4%
Any criteria	304,308	23.4%	292,767	23.0%	11,541	43.0%

[a] Components of hypertension criteria: "Diagnosis code only" refers to having ≥ 1 hypertension diagnosis code in 2022; "High BP only" refers to having ≥ 2 high BP at ≥ 130/80 mmHg in 2022; "Medication only" refers to having ≥ 1 prescription of antihypertensive medication in 2022; "Any criteria" refers to meeting any of the above 3 criteria.

[b] Spironolactone is the only medication on the antihypertensive medication list that could have been prescribed for PCOS. Spironolactone was approved by the Food and Drug Administration for hypertension treatment, and could be used "off-label" to manage the symptoms of PCOS.

AEMR-US, IQVIA Ambulatory Electronic Medical Record-US; BP, blood pressure; PCOS, polycystic ovary syndrome
